# Special Issue—The Conformational Universe of Proteins and Peptides: Tales of Order and Disorder

**DOI:** 10.3390/molecules26123716

**Published:** 2021-06-18

**Authors:** Marilisa Leone

**Affiliations:** Institute of Biostructures and Bioimaging (IBB), National Research Council of Italy (CNR), Via Mezzocannone 16, 80134 Naples, Italy; marilisa.leone@cnr.it

Among biological macromolecules, proteins hold prominent roles in a vast array of physiological and pathological processes. The protein sequence-structure-function paradigm establishes that the amino acid sequence governs the structure that in turn determines the function [1]. Thus, the knowledge of the 3D structure of a protein, along with the possible conformational transitions occurring upon interaction with diverse ligands, are essential to fully comprehend its biological function.

Apart from globular well folded proteins, during the past years intrinsically disordered proteins (IDPs) have attracted a lot of attention. IDPs present a general tendency to aggregate and may form toxic amyloid fibers and oligomers associated with many human pathologies [2,3,4]. Intensive studies have been lately devoted to structural characterizations of aggregates formed by IDPs, along with the search for novel techniques to easily perform such analyses. Interestingly, IDPs are also known to often undergo a disorder to order switch following binding to their targets that generates specific outcomes in a cellular context.

Peptides, which are characterized by a smaller size than proteins, represent key elements of cells as well. Peptides can even find diagnostic and therapeutic applications, for instance working as tumor markers [5]. In the drug discovery field, the structural features of bioactive peptides are employed to design potential novel drugs acting as selective modulators of specific receptors or enzymes. Nevertheless, synthetic peptides reproducing different protein fragments have frequently served as model systems in folding studies relying on structural investigations in water and/or other environments.

This Special Issue comprehends contributions (i.e., seven original research articles and five reviews) on the above-described topics and, in detail, it includes structural studies on globular folded proteins, IDPs and bioactive peptides. These works were conducted by utilizing different experimental (including solution, solid states and high-pressure NMR and mass spectrometry) and/or computational approaches (mainly molecular dynamics simulations and bioinformatic tools).

A broad range of structural biology topics are covered by the Special Issue as summarized below.

Proteins under biological conditions unfold and refold several times in vivo showing a marginal structural stability. A detailed molecular-level knowledge not only of the native but also of the diverse non-native conformational states, which are accessible to a protein in solution (i.e., its denatured state ensemble (DSE)), is necessary to fully comprehend its function. Several investigations employed short peptides as models to obtain the canonical features of the DSE [6]. Short peptides are advantageous within this context as, being too short to assume a compact fold, they can sample unfolded states under folding conditions. Different peptide structural studies showed the strong tendency for the polyproline II (PPII) backbone conformation, which consequently could be a dominant component of the DSE [6]. Another structural model for the DSE is represented by the protein coil library that was built up from the segments of protein structure in the Protein Data Bank (PDB) that are located outside the α-helix and β-strand domains. Overall, coil libraries exhibit structural trends that are in good agreement with the results from peptide structural studies underlying a high preference for PPII that can be also linked to the amino acid type. In order to assess structural preferences in unfolded states under non-denaturing conditions, IDPs can also be employed as an additional experimental model system. An interesting review by Steven T. Whitten and collaborators critically analyzes spectroscopic and calorimetric works on short peptides, structures in the protein coil library and sequence and temperature-based investigations of IDP hydrodynamic sizes; they demonstrate how the three model systems used for describing unfolded proteins under folding conditions deliver a consistent structural and energetic view of the DSE [6]. Results from analyses of the three model systems (i.e., peptides, the coil library and IDPs) highlight that the structural and energetic features of the DSE at normal temperatures can be predicted by a PPII-dominant ensemble. At cold temperatures, the DSE can undergo a transition in population toward the *α*-helix backbone conformation as revealed by the analyses of both peptides and IDPs.

The Special Issue further includes interesting reviews describing structural and functional features of different proteins. Leonardo L. Fruttero and collaborators focus on intrinsically disordered polypeptides from plant ureases (i.e., Jaburetox and Soyuretox). Jaburetox represents a recombinant peptide derived from the jack bean (*Canavalia ensiformis*) urease that possesses entomotoxic and antimicrobial functions [7]. NMR studies point out that Jaburetox possess only low amounts of secondary structure and behaves as an IDP. Nevertheless, Jaburetox can undergo a disorder to order transition after binding lipid membranes. Soyuretox is another IDP homologous to Jaburetox and it is derived from the soybean (*Glycine max*) ubiquitous urease. Compared to Jaburetox, Soyuretox contains a higher secondary structure content but preserves similar entomotoxic and fungitoxic properties. Due to the positive toxicity profile, both peptides find biotechnological applications and have in fact been already used to generate transgenic crops giving rise to plants active against insects and nematodes [7].

Nicholas J. Bradshaw and collaborators focus, instead, on the TRIOBP (TRIO and F-actin Binding Protein) isoforms and describe within their review the proteins structural characteristics along with their function in actin stabilization and the relationship to pathological conditions (deafness, mental illness and cancer) [8]. In detail, the TRIOBP gene encodes multiple proteins, TRIOBP-1 represents principally a structured protein that interacts with F-actin and avoids its depolymerization. TRIOBP-1 has been linked to schizophrenia, as it can give rise to protein aggregates in the brain. TRIOBP-4 is, on the other hand, a completely disordered protein and a few of its mutations are related to severe or profound hearing loss. TRIOBP-1 and TRIOBP-4 have both been related to cancer [8].

Another interesting review by Christian Roumestand and collaborators is centered on High-Pressure (HP)-NMR. The authors summarize recent advances of HP-NMR and describe how this technique can be employed to characterize, at a quasi-atomic resolution, the protein folding energy landscape [9]. Globular proteins can be perturbed in several ways and high-hydrostatic pressure represents an alternative destabilizing method. At difference from heat or chemical denaturant, which generate a uniform protein destabilization, pressure produces local effects on protein regions or domains provided with internal voids. HP-NMR spectroscopy allows one to follow the structural transitions occurring upon unfolding and to study the kinetic properties of the process [9].

Beat H. Meier, Anja Böckmann and collaborators describe within their review the lessons learned from studies on two ATPases (the bacterial DnaB helicase from *Helicobacter pylori* and the multidrug ATP binding cassette (ABC) transporter BmrA from *Bacillus subtilis*) [10]. The authors report on NMR approaches that can be employed to examine proteins binding to ATP-mimics. In order to reveal conformational and dynamic changes occurring upon interaction with ATP-mimics, carbon-13, phosphorus-31 and vanadium-51 solid-state NMR spectra of the proteins or the bound molecules are shown.

The reported information can surely be relevant to researchers conducting studies on other NTPases (Nucleoside TriPhosphatases) [10].

An interesting research article by Michael O. Glocker and collaborators highlights how mass spectrometry techniques have enlarged their horizons by becoming additional tools to study intermolecular interactions [11]. In detail, the authors report on an original approach relying on electrospray mass spectrometry (called ITEM-TWO (Intact Transition Epitope Mapping-Thermodynamic Weak-force Order)) that requires only a small sample amount and permits the simultaneous identification of epitopes (such as peptide segments that are recognized by a certain antibody) and gas phase binding strengths for the antibody–epitope peptide interactions [11].

In this pandemic era, most research efforts are centered on SARS-CoV-2 related studies. Thus, a contribution to the field is also included in this Special Issue. Vladimir N. Uversky and collaborators, by utilizing different bioinformatic tools, analyze amino acid sequences of ACE2 (Angiotensin-converting enzyme 2) receptors from eighteen non-human species and compare them with the human ACE2 sequence pointing out the degree of variability [12]. Results indicate that many non-human species have, in the binding site of ACE2 receptor, similar amino acid types to humans letting speculate that the RBD (Receptor Binding Domain) of the SARS-CoV-2 Spike (S) protein could be involved in similar interactions with ACE2 receptors from different species. Consequently, this detailed investigation of the ACE2 protein let speculate that, indeed, interspecies SARS-CoV-2 transmission could be quite possible and allows to formulate a possible transmission flow. Nevertheless, the authors also examine the per-residue intrinsic disorder tendency of the ACE2 proteins from several species pointing out a certain degree of similarity among disorder profiles and highlighting regions where the most striking differences are evident [12].

Authors Alberto Perez and Lijun Lang report on the p53-MDM2 (Mouse Double Minute 2 homolog) interaction, which is highly relevant in cancer research. In fact, p53 triggers programmed cell death when cells misbehave, while MDM2 downregulates p53 anticancer activity; inhibitors of the p53-MDM2 interaction thus possess anticancer potentials [13]. The authors present, in their research article, a computational approach that could result really useful for drug design. Nowadays computational tools such as virtual screening techniques have to face a huge challenge when dealing with the binding of flexible peptides that could fold following interaction to specific receptors [13]. The authors in their research article investigate the binding of five peptides, including three intrinsically disordered ones, to MDM2 with a Bayesian inference approach (MELDXMD (Modeling Employing Limited Data Accelerated MD)). The method is able to capture the folding upon binding mechanism, showing the most likely bound conformations and highlighting the differences in the binding mechanisms [13].

Work by Rajni Verma, Jonathan M. Ellis and Katie R. Mitchell-Koch focuses instead on molecular dynamics simulations of the enzyme YqhD. YqhD represents an *E. coli* alcohol/aldehyde oxidoreductase that, beginning from a wide range of materials, is able to generate relevant bio-renewable fuels and fine chemicals [14]. The computational work sheds light on the conformational dynamics of the enzyme upon interaction with oxidized and reduced NADP/H cofactor [14]. The study highlights how YqhD complexed with NADP may fluctuate between open and closed conformations, while interaction with NADPH induces a slower opening/closing dynamic of the cofactor-binding site. This dynamical view let speculate that the frequent opening of the binding cleft is necessary to favor release of NADP, while a more closed conformation is necessary to enhance NADPH interaction along with aldehyde reductase activity [14]. This work clearly points out how molecular dynamics simulations may provide access to structural details that could help better understand how enzymes work.

In their research article, Richard P. Cheng and collaborators analyze, largely by using NMR analyses, cross-strand lateral ion-pairing interactions and their importance for antiparallel β-sheet stability [15]. The authors provide interesting insights for the design of functional peptides provided with lateral ion-pairing interactions across antiparallel-strands. In detail, they perturbed the cross-strand lateral ion-pairing interactions in a β-hairpin peptide by swapping the position of ammonium and carboxylate containing residues with different side-chain lengths [15]. Chemical shift data permit gaining the fraction folded population and folding free energy. Results point out that, similarly to the unswapped peptide, the most stabilizing cross-strand contacts occur between short residues, although an increase in folded populations upon swapping is detected [15].

David A. Snyder and collaborators tells us about protein flexibility and its importance for proper protein functioning by performing a comparative investigation of protein flexibility captured by crystallographic B-factors, molecular dynamics and NMR studies [16]. This work highlights that NMR- structural ensembles present a pattern in the coordinate uncertainties of backbone heavy atoms that also recurs in coordinate variances across MD trajectories but not in crystallographic B-factors [16]. This evidence let speculate that either MD trajectories and NMR structures are able to detect the motional behavior of peptide bond units not contemplated by B-factors or could highlight a deficit linked to the force fields employed in both NMR and MD calculations [16].

Finally, Gennaro Esposito and collaborators in their research article report on the structure characterization of a nanobody by utilizing solution state NMR techniques coupling to molecular modelling investigation [17]. Nanobodies derive from heavy chain-only antibodies, which can be found in camelids, with their smaller molecular size and higher stability represents an alternative to mAbs for therapeutic use. Two nanobodies, Nb23 and Nb24, are able to block similarly self-aggregation of highly amyloidogenic variants of *β*2-microglobulin. The authors carried out a structural characterization of Nb23. The study points out peculiar structural features of Nb23 with respect to Nb24, which could be linked to diverse target antigen affinity [17].

In conclusion, this ensemble of studies clearly stresses how the knowledge of structural features enables the understanding of the multifaceted roles of protein and highlights the importance of flexible regions and disorder in dictating binding events and directing protein functions under normal physiological and pathological conditions. Coupling of experimental and computational work is always necessary to reach a complete and detailed structural portrait of a protein in its isolate state and when it is bound to a ligand (intended either as a small cofactor or a bigger peptide). Researchers are still eager to develop better computational tools to keep into account proteins/peptide flexibility and to predict interactions of proteins with large flexible systems, such as peptides, and in the next few years much efforts will likely be devoted towards reaching this goal and optimizing existing tools. Nevertheless, the reported studies also highlight the continuous search by the scientific community for novel improved experimental techniques to a protein/peptide structure, dynamics and interactions.

Given the variety of topics embraced by this Special Issue, a great interest from researchers working in the protein/peptide structural biology field is expected.
